# Reply to ‘Oxic methanogenesis is only a minor source of lake-wide diffusive CH_4_ emissions from lakes’

**DOI:** 10.1038/s41467-021-21216-1

**Published:** 2021-02-22

**Authors:** Marco Günthel, Daphne Donis, Georgiy Kirillin, Danny Ionescu, Mina Bizic, Daniel F. McGinnis, Hans-Peter Grossart, Kam W. Tang

**Affiliations:** 1 Department of Environmental Microbiology and Biotechnology, University of Warsaw, Warsaw, Poland; 2 Department of Experimental Limnology, Leibniz Institute of Freshwater Ecology and Inland Fisheries, Stechlin, Germany; 3 Department of Biosciences, Swansea University, Swansea, UK; 4 Aquatic Physics Group, Department F.-A. Forel for Environmental and Aquatic Sciences, Faculty of Science, University of Geneva, Geneva, Switzerland; 5 Department of Ecohydrology, Leibniz Institute of Freshwater Ecology and Inland Fisheries, Berlin, Germany; 6 Institute of Biochemistry and Biology, Potsdam University, Potsdam, Germany

**Keywords:** Biogeochemistry, Limnology

**Replying to** F. Peeters & H. Hofmann *Nature Communications* 10.1038/s41467-021-21215-2 (2021)

The prevailing paradigm in methane research is that biological methane production is exclusive to anoxic or near-anoxic habitats such as sediments and oxygen-deficient bottom waters in lakes. Paradoxically, methane supersaturation in oxic lake waters is widely reported. To resolve this paradox while preserving the paradigm, some researchers assume this methane originates entirely from anoxic sources and is then transported to the oxic waters through physical processes^1–3^. However, multiple recent studies have repeatedly shown, methane production can and does occur under oxic conditions on land, in the seas and in freshwaters, driven by diverse organisms within different life domains (Table 1 and references therein) and via photochemical conversion^4^. These findings raise legitimate questions about the nature of the environmental dynamics and global budget of methane. Because oxic methane production (OMP) is a recent discovery, its contribution to atmospheric emission is unknown. We conducted a whole-lake basin methane mass balance and analysed relevant literature data to estimate the contribution of OMP to surface emission versus lake morphometry.

**Table 1:** Literature examples of oxic methane production (OMP) in different habitats and by different domains of life.

Organism	Domain	CH_4_ production rate	Evidence	Reference
*TERRESTRIAL*				
Plants	Eukaryote		INC, ISO	Keppler et al. (2006)^20^
Plants	Eukaryote		INC	Messenger et al. (2009)^21^
Methanogens	Archaea		INC, ISO, OMIC	Angel et al. (2011)^22^
Fungi	Eukaryote		INC, ISO	Lenhart et al. (2012)^23^
Plants	Eukaryote		INC, ISO	Althoff et al. (2014)^24^
Methanogens	Archaea		MB, OMIC, PHYS	Angle et al. (2017)^14^
Cyanobacteria	Prokaryote		INC, ISO	Bizic et al. (2020)^15^
*MARINE*				
Mixed assemblage			INC, OMIC	Karl et al. (2008)^17^
Bacteria	Prokaryote		INC, STAT	Damm et al. (2010)^25^
Cyanobacteria	Prokaryote		INC	White et al. (2010)^26^
α-Proteobacteria	Prokaryote		INC, OMIC	Carini et al. (2014)^27^
Haptophytes	Eukaryote		INC, ISO	Lenhart et al. (2016)^28^
Bacteria	Prokaryote		INC, OMIC	Repeta et al. (2016)^29^
Haptophytes	Eukaryote		INC, ISO	Klintzsch et al. (2019)^30^
Cyanobacteria	Prokaryote		INC, ISO	Bizic et al. (2020)^15^
γ-Proteobacteria	Prokaryote		INC	Ye et al. (2020)^32^
Haptophytes	Eukaryote		INC	Klintzsch et al. (2020)^31^
*FRESHWATER*				
Methanogens, algae	Archaea, Eukaryote	38–58 nmol l^−^^1^ day^−1^ (Lake Stechlin)	INC	Grossart et al. (2011)^11^
Methanogens, algae	Archaea, Eukaryote	210–240 nmol l^−^^1^ day^−1^ (Lake Cromwell)	ISO, MB	Bogard et al. (2014)^33^
α-, γ-proteobacteria	Prokaryote		INC, OMIC	Yao et al. (2016)^13^
Mixed assemblage		110 nmol l^−^^1^ day^−1^ (Lake Hallwil)	MB	Donis et al. (2017)^7^
γ-Proetobacteria	Prokaryote	0.2–0.7 nmol l^−^^1^ day^−1^ (Yellowstone Lake)	INC, ISO, OMIC	Wang et al. (2017)^34^
Mixed assemblage			ISO, MB, PHYS	DelSontro et al. (2018)^35^
Proteobacteria	Prokaryote	54–257 nmol l^−^^1^ day^−1^ (Lake Bonney)	INC, OMIC	Li et al. (2019)^36^
Cyanobacteria	Prokaryote		INC, OMIC	Khatun et al. (2019)^37^
Mixed assemblages		72–88 nmol l^−1^ day^−1^ (Lake Stechlin) 78–138 nmol l^−^^1^ day^−1^ (Lake Hallwil)	MB	Günthel et al. (2019)^39^
Cyanobacteria	Prokaryote		INC, ISO	Bizic et al. (2020)^15^
Cyanobacteria	Prokaryote		STAT	Khatun et al. (2020)^38^
Green algae, diatoms, cryptophytes	Eukaryote	50–210 nmol l^−^^1^ day^−1^ (Lake Stechlin)	INC, ISO, MB, STAT	Hartmann et al. (2020)^18^
Picoeukaryotes, diatoms	Eukaryote		STAT	Leon-Palmero et al. (2020)^41^
Proteobacteria	Prokaryote	24–547 nmol l^−1^ day^−1^ (5 Lakes)	INC, ISO, OMIC	Perez-Coronel and Beman (2020)^42^

OMP evidence type: *INC* incubation experiments, *ISO* isotope techniques, *MB* mass balance approaches, *OMIC* molecular biological methods, *PHYS* physical modelling, *STAT* statistical analyses.

OMP has been observed in different limnic systems, e.g. temperate and arctic regions (DelSontro et al. 2018^35^, Li et al. 2019)^36^, high-elevation (Perez-Coronel and Beman, 2020)^42^, and throughout the oligo-to-eutrophic nutrient spectrum (DelSontro et al., 2018^35^, Khatun et al., 2020^38^, Ye et al., 2020)^32^.

Because the dynamics of methane concentration and isotope signal in lake waters are influenced by different and opposing processes simultaneously, one cannot meaningfully deduce the presence or absence of OMP without properly accounting for modulations by physical and biological processes. For example, underestimating surface emission or ignoring oxidation would lead to incorrect interpretation of methane concentration and isotope data and incorrect dismissal of OMP (Supplementary Note 1).

By balancing the gains and losses of epilimnetic methane in a stratified water column, we estimated the contribution of oxic versus anoxic methane to surface emission (Supplementary Fig. 1). Epilimnetic methane may originate from lateral and vertical transport from anoxic zones, ebullition, and internal oxic production (OMP); surface emission and oxidation are the loss terms.

Surface methane emission can be measured directly using a flux chamber, or, in the absence of direct measurements, it is often modelled from surface-water methane concentrations and wind speeds. Both methods are commonly used but the results can differ considerably, and there exist many different wind-based models (for a more detailed discussion we refer readers to the literature^5,6^). Notably in their manuscript, Peeters and Hofmann excluded our direct measurements of methane fluxes to the atmosphere and exclusively rely on modelling approaches (Supplementary Note 2). We instead combined direct measurements with models that were established for the target lake. Therefore, we consider that our direct measurement approach minimises methodological and model biases, and better represents reality.

For Lake Hallwil, we used the littoral sediment-to-water methane flux as determined by Donis et al.^7^ who implemented two littoral sediment core measurements sampled at 3 and 7 m depth and applying Fick’s law. In contrast, Peeters and Hofmann implemented only the upper sediment core into their re-analysis. They justify this choice by stating the cores’ methane isotope signature vary. As the depth of Lake Hallwil’s surface mixed layer increased over the seasonal progression^7^, both sediment cores should be considered in the mass balance especially in the light of natural variability. For Lake Stechlin, we used data from two mesocosms and the open-water to resolve littoral methane input (Supplementary Notes 3 and 4). We estimated ebullitive methane fluxes as negligible in Lake Stechlin^8,9^. We further applied an ebullitive flux of 1.2 ± 0.8 mmol m^−2^ d^−1^ to Lake Hallwil^10^, giving a total sediment methane input of 3 mmol m^−2^ d^−1^ when combined with the diffusive flux, which is higher than the value assumed by Peeters and Hofmann. Vertical diffusive input was calculated from empirically measured methane concentration profiles and turbulent diffusivities. We parameterised methane oxidation as 30% of internal production for Lake Stechlin; in a sensitivity analysis, we evaluated this assumption and also considered the most conservative scenario, e.g., OMP set to minimum. For Lake Hallwil, methane oxidation rates were measured by experiments.

By balancing the different input and output fluxes, we produced the first system-wide OMP estimate for Lake Stechlin, which agrees well with direct bottle incubation measurements reported earlier^11^. To further account for (seasonal) variabilities and measurement uncertainties, we conducted Monte Carlo simulations and sensitivity analysis applying various conservative scenarios to the mass balance. It is, however, worth noting that the mass balance is sensitive to the flux parameterisation and the accuracy of its result is hinged on how reliably one accounts for these fluxes. To better resolve OMP and allow for more general and firm statements about OMP (including different lake systems), future studies should aim to reduce uncertainties associated with the littoral methane input (e.g. methodological uncertainty in sediment core measurements^12^) and methane oxidation—two key parameters in the epilimnetic methane budget.

OMP by diverse organisms (Table 1) and pathways^13–15^ point to its wider potential relevance on a global scale. To examine how OMP may vary according to lake characteristics, we combined our results with analysis of literature data to estimate OMP contribution in relation to basin morphometry (Supplementary Note 5). The epilimnetic methane sources considered here are littoral sediment and OMP. On a whole-system level, the relative contributions of these sources are proportional to the total littoral sediment area and the epilimnion volume, respectively. Because the ratio of littoral sediment area to epilimnion volume decreases with increasing lake size, the contribution of OMP to surface emission is expected to increase with lake size. This trend does not change even when we assume a larger littoral sediment area by decreasing the sediment slope as suggested by Peeters and Hofmann (Fig. 1). As the current OMP dataset is limited to only a few lakes (four data points based on mass balance and seven based on transport modelling), future studies should aim to increase the number and types of lakes to verify the trend on a larger scale.

**Fig. 1:**
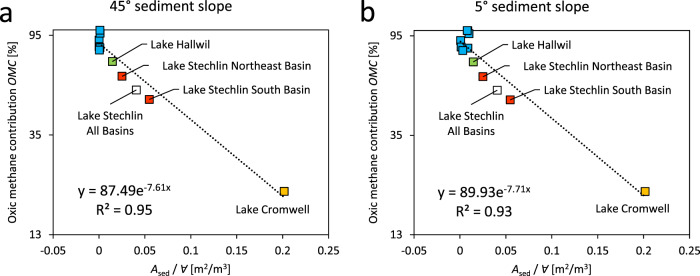
Oxic methane contribution (OMC) to surface emission in relation to lake morphology. Comparison of (**a**) the original relationship and (**b**) the alternative parameterisation using a smaller sediment slope angle. *A*_sed_ is the littoral sediment area and *∀* is the surface mixed layer volume. Note, OMC is defined as in our original study; the *x*-axis is linearly scaled, and the *y*-axes is scaled to log_2.7_.

Note, as Peeters’ and Hofmann’s re-analysis excludes internal methane modulation, their OMP estimates reflect net rates while our study presents gross rates. Accordingly, their contribution pattern of oxic versus anoxic methane source to surface emission (NOMC) cannot be directly compared to our estimates (OMC) (further discrepancy is explained by Supplementary Note 5).

Oxic methane production defies the century-old teaching of anoxic methanogenesis and the convention of considering only anoxic sources in methane research; as such, skepticism is expected. While some may dismiss OMP as irrelevant^16^, others take a more practical approach and investigate the phenomenon at the ecological, organismal, and molecular levels^13,17^. However, the novelty of OMP also means researchers are still trialling different methods, each with their limitations (Table 2).

**Table 2:** Overview on approaches to investigate oxic methane production (OMP) in lake waters.

Approach	Description	Caveats	Reference examples
Incubation of • Lake water • Enrichment cultures	Cultivating microbes in closed containers and recording CH_4_ concentration over time. Additionally, the change in ^13^C/^12^C carbon isotope ratio in dissolved methane can be measured.	Bottle enclosure may alter the light and nutrient conditions versus in situ. Long-term incubations (exceeding hours) may not reflect in situ conditions due to changes to the production-consumption equilibrium (e.g., nutrient depletion, community alterations).	Grossart et al. (2011)^11^, Bizic et al. (2020)^15^, Günthel et al. (2020)^40^, Hartmann et al. (2020)^18^, Klintzsch et al. (2019, 2020)^30,31^
Metagenomics	Molecular analysis of relevant enzyme machinery or genes.	Qualitative evidence. Presence of relevant genes and enzymes indicates production potential, but actual production rate can be affected by inhibitors, missing precursors, unfavourable conditions, epigenetic modulation, etc.	Carini et al. (2014)^27^, Yao et al. (2016)^13^, Perez-Coronel and Beman (2020)^42^
Statistical analysis	Methane concentration is measured together with other lake parameters. Statistical models are applied to test for correlative significance and predictive power.	Individual methane sources and sinks can be overlooked due to the complex lake water methane cycling. Results lack mechanistic understanding of the underlying processes.	Fernandez et al. (2016)^3^, Günthel et al. (2020)^40^, Khatun et al. (2020)^38^, Leon-Palmero et al. (2020)^41^
Physical modelling	Combining physical mechanistic aspects with correlative analysis.	Underrepresentation of internal biological modulation (oxidation and OMP).	Peeters et al. (2019)^16^
Mass balance of epilimnion in • Whole-lake basin or • Mesocosms/enclosures	Methane input and output fluxes for the epilimnion are experimentally determined and balanced. Discrepancy is attributed to OMP.	Accuracy of OMP production rates depends on how reliably methane fluxes have been determined. Spatio-temporal data resolution is often limited.	Bogard et al. (2014)^33^, Donis et al. (2017)^7^, Günthel et al. (2019)^39^, Peeters et al. (2019)^16^, Hartmann et al. (2020)^18^
Methane isotope analysis • Comparing ambient signatures or • Isotope budgets	Analysing carbon (and hydrogen) stable isotope signatures of methane sources and considering isotope fractionation by biochemical and physical reactions (e.g., oxidation, OMP, phase exchange). Analogue to mass balance.	This analysis requires knowing (i) the quantity of all mass fluxes, (ii) isotope characteristics of all methane sources, (iii) isotope fractionation by biochemical and physical processes. Different precursors and biochemical production/consumption pathways can result in different isotope signatures.	Tang et al. (2014)^9^, DelSontro et al. (2018)^35^, Günthel et al. (2020)^40^, Hartmann et al. (2020)^18^, Tsunogai et al. (2020)^19^

A better understanding of production, storage, consumption, and distribution processes of methane, including methane produced in oxic environments, is needed to improve the assessment of the global methane cycle. This requires better spatio-temporal data resolution and better constraints of data uncertainties by using multiple methods. For instance, OMP rates determined by bottle incubations can complement results based on mass budgets, as we did in our study. The incorporation of methane carbon^18^ and hydrogen^19^ isotope data into mass budgets is a promising way to further tease apart the different methane sources. Omic approaches can be used to investigate the different OMP pathways and the organisms involved.

We have discussed the caveats of our mass balance analysis, such as the limited amount of OMP and littoral flux data, limited types of lakes being considered, and the influence by other compounding factors. The global significance of OMP can only be fully assessed when more relevant data become available, but this also requires researchers to look beyond the anoxic paradigm and consider OMP in future methane measurements. We hope our and others’ work will continue to stimulate more research and constructive discussions on this topic.

## Supplementary information

Supplementary Information

## Data Availability

Data are made available in graphical or tabular form throughout the paper and Supplementary Information. Source data are provided with this paper.

## Source data

Source Data
