# Supplementary material for: Reply to ‘Oxic methanogenesis is only a minor source of lake-wide diffusive CH4 emissions from lakes’
Source: Nat Commun. 2021 Feb 22;12:1205. doi: 10.1038/s41467-021-21216-1 (PMC7900099; doi:10.1038/s41467-021-21216-1)
Supplement: Supplementary file 1 — Supplementary Information [file 41467_2021_21216_MOESM1_ESM.pdf]

SUPPLEMENTARY INFORMATION for

Reply to ‘Oxic methanogenesis is only a minor source of lake-wide diffusive CH4 emissions from lakes’

by Günthel et al.

Supplementary Note 1:

Anoxic methanogenesis has been the paradigm in methane research for generations; questions about the novel idea of oxic methane production are expected and welcomed. Our paper acknowledges dissenting opinions in the literature including Peeters et al. 2019.

Peeters and Hofmann cited Peeters et al. as what they consider as a more valid mass balance analysis of methane dynamics in Lake Hallwil. However, Peeters et al.'s mass balance model *"considers as source of CH_4 diffusive fluxes from the sediments, loss of CH_4 due to diffusive emissions from the water surface to the atmosphere, ...and lateral transport of CH_4 by turbulent mixing within the surface mixed layer"*; hence, their mass balance excludes open-water biogeochemical processes such as methane oxidation, and is therefore inadequate (e.g. systematic discount of oxic methane production (OMP)). Likewise, Peeters' and Hofmann's interpretation of isotope data in Donis et al. 2017 is inadequate without proper consideration of isotopic shifts caused by physical (emission phase change) and biological processes (oxidation and OMP). Note that we did not use the isotope data for our mass balance analysis (see further discussion in Supplementary Note 5).

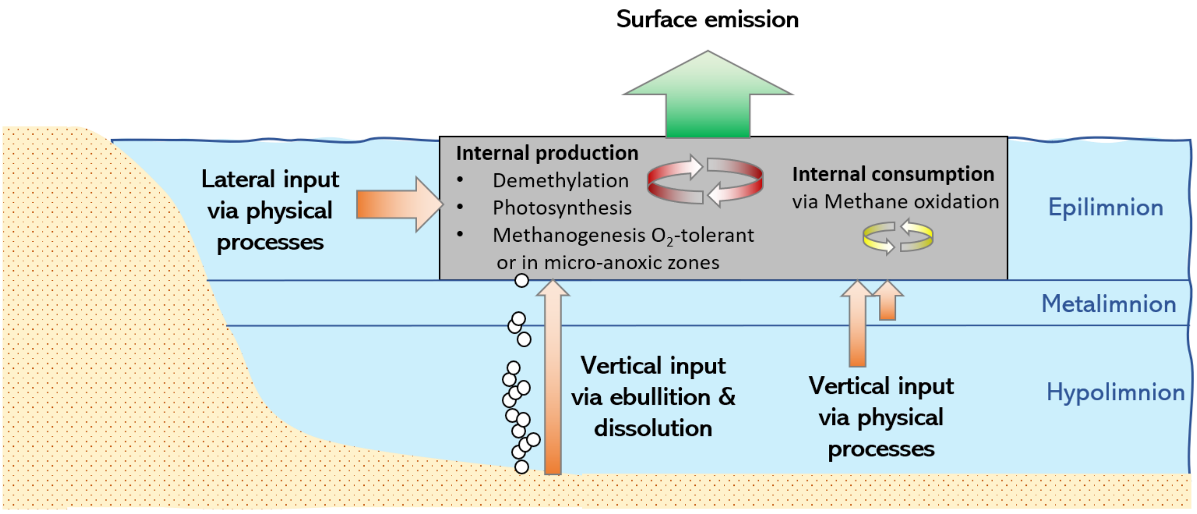

Supplementary Figure 1. Epilimnetic methane mass balance. *The mass balance approach compares physical methane transport (lateral, vertical) and output fluxes (surface emission, oxidation) in epilimnetic waters. The difference between input and output fluxes is attributed to oxic methane production.*

Supplementary Note 2:

Peeters and Hofmann alleged that Donis et al. 2017 and thence Günthel et al. 2019 overestimated surface emissions in Lake Hallwil. The emissions reported in Donis et al. were measured directly with flux chamber (see McGinnis et al. 2015) and therefore it was not necessary to calculate gas transfer constants (k_{600}), as Peeters and Hofmann claimed. These

direct emission measurements were also excluded from Peeters' and Hofmann's re-analysis. Supplementary Table 1 exemplarily displays the conversion of flux chamber readings to methane emission values which are listed by Supplementary Table 2 (mean±SD 0.7±0.4 mmol m^{-2} d^{-1}).

Supplementary Table 1. *Exemplary conversion of flux chamber readings to methane surface emission.*

Air Pressure	Water Temperature MEAN	Slope CH_4 (from LGR)	Chamber Temperature MEAN	Chamber volume	Bottom area of chamber	R-gas constant	Chamber Temperature	CH_4 Flux	CH_4 Flux
[atm]	[°C]	[ppm s^{-1}]	[°C]	[mL]	[m^2]	[mL atm $K^{-1}mol^{-1}$]	[K]	[mol m^{-2} d^{-1}]	[mmol m^{-2} d^{-1}]
1	20.5	0.0013	28	16755.72	0.126	82.0562	301.15	0.00060	0.604
1	20.5	0.0015	28	16755.72	0.126	82.0562	301.15	0.00070	0.697
1	20.5	0.0011	28	16755.72	0.126	82.0562	301.15	0.00051	0.511

Supplementary Table 2. *Individual methane emission values retrieved from flux chamber measurements.*

Date	Station	CH_4 Flux [mmol m^{-2} d^{-1}]	Date	Station	CH_4 Flux [mmol m^{-2} d^{-1}]
11-Jun-15	Hallwil St 8b (wp 382)	0.604	25-Jun-16	Sta 8	0.404
11-Jun-15	Hallwil St 8b (wp 382)	0.697	25-Jun-16	Sta 8	0.665
11-Jun-15	Hallwil St 8b (wp 382)	0.604	25-Jun-16	Sta 8	0.656
11-Jun-15	Hallwil St 8b (wp 382)	0.511	25-Jun-16	Sta HW1	0.778
12-Aug-15	Diffuser	0.827	25-Jun-16	Sta HW1	0.780
12-Aug-15	Diffuser	1.147	25-Jun-16	Sta HW1	1.216
12-Aug-15	Diffuser	1.434	25-Jun-16	Sta HW1	0.902
14-Apr-15	Sta 8	0.519	06-Jul-16	Sta HW 8	0.909
14-Apr-15	Sta 8	0.649	06-Jul-16	Sta HW 8	1.011
14-Apr-15	Sta HW1	0.386	06-Jul-16	Sta HW 8	0.883
14-Apr-15	Sta HW1	0.643	06-Jul-16	St HW 483 (north)	1.412
15-May-15	Sta 8	0.028	06-Jul-16	St HW 483 (north)	1.072
15-May-15	Sta 8	0.032	06-Jul-16	St HW 483 (north)	0.839
15-May-15	Sta 8	0.030	06-Jul-16	Sta HW1 (south)	1.248
15-May-15	Sta 8	0.029	06-Jul-16	Sta HW1 (south)	0.761
15-May-15	Sta HW1	0.297	06-Jul-16	Sta HW1 (south)	1.262
15-May-15	Sta HW1	0.220	06-Jul-16	Sta HW8	0.433
15-May-15	Sta HW1	0.287	06-Jul-16	Sta HW8	0.264
15-May-15	Sta HW1	0.343	06-Jul-16	Sta HW8	0.674
25-Jun-16	Sta 8	0.280			

Supplementary Note 3:

We used both mesocosm data sets when estimating the littoral sediment flux, as indicated by the mean value and standard deviation in Günthel et al. 2019. The result was in fact 50% higher than the more recent direct sediment-core measurement (Hartmann et al. 2020), meaning we may have overestimated the littoral methane contribution. While Peeters and Hofmann question the comparison to Hartmann et al.'s measurements (e.g. *"It seems unrealistic that such low sediment fluxes are representative for the average CH_4 flux from littoral sediments in the South Basin of Lake Stechlin."*), there are independent studies indicating generally low methanogenesis activity in Lake Stechlin (Casper et al. 2003, Casper et al. 2005, Conrad et al. 2007), and that the majority happens below 20 cm of depth (Casper 1996), potentially explaining the low fluxes at the sediment-water interphase reported by Hartmann et al. (note, methane oxidation can efficiently remove the majority of methane).

Further, the mean and standard deviation of our littoral sediment flux estimate were incorporated in the Monte Carlo simulation to account for data uncertainties when computing OMP rates. When claiming that we underestimated the littoral sediment flux Peeters and Hofmann rely on the relationship between energy dissipation (ε) and the gas transfer constant (k_{600}). While parameterising this relation as $k_{600} \sim \varepsilon^{1/4}$, Peeters and Hofmann missed to mention that the parameterisation of this relation is subject to ongoing research and that currently, there is no consensus which parameterisation produces more accurate values. Further, we did not deploy an outdated approach to deduce Kρ from shear microstructure (e.g. Gregg et al. 2018) which we additionally refined by implementation of the law-of-the-wall in the unstratified epilimnion following Kirillin et al. 2012.

Peeters and Hofmann implied that we assumed sediment methane flux in Lake Stechlin was independent of temperature. Incorporating a temperature dependency of the littoral methane flux was unnecessary because we quantified the littoral methane flux in August at the same water temperature as it was in June and July (20°C) (see Supplementary Fig. 3 in Günthel et al. 2019). By applying the same value to May (<20°C), we likely had overestimated the littoral methane input. Note, we referenced the temperature dependency in the method section of our original study Günthel et al. 2019.

Instead of presenting relevant data or analysis for Lake Stechlin, Peeters and Hofmann simply mentioned the paper by Yvon-Durocher et al. Below is Fig. 2a from Yvon-Durocher et al. 2014 showing *"temperature dependence of CH_4 emissions at the ecosystem level"*:

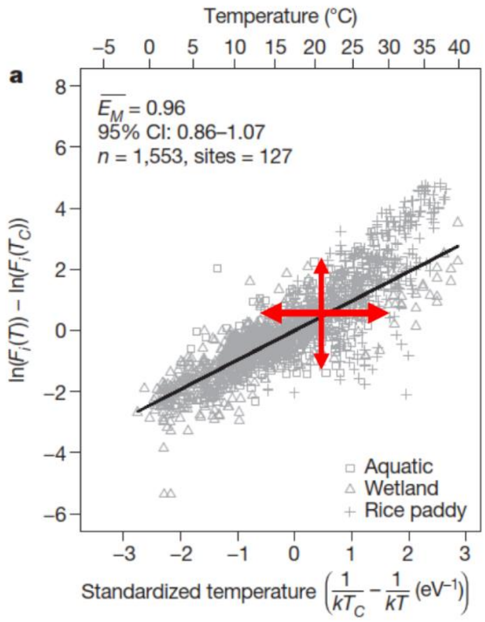

Figure 2a *from Yvon-Durocher et al. 2014; red arrows are added by us to illustrate the data variability around the regression line at 20°C (approx. littoral temperature in Lake Stechlin). Note the y-variable is not the actual emission, but the difference between emission at absolute temperatures and emission at some average temperatures.*

While methane emission is temperature dependent, there is a large amount of scatter around the general trend line in both x and y directions (red arrows added by us). Lake Stechlin surface mixed water temperature above the littoral was ca. 20°C (Supplementary Fig. 3 in Günthel et al. 2019). According to Yvon-Durocher et al. 2014 Fig. 2a, the corresponding y-value varies between 2.2 and -1.8 on a natural log scale, which translates to a >50-fold difference between the actual upper and lower values. Likewise, the same y-value could correspond to a temperature between 13°C and 30°C. We also refer readers to the Extended Data Figure 2 in Yvon-Durocher et al. "*Correlations of average site temperatures with average CH_4 emissions and CH_4 emissions at fixed temperature for globally distributed ecosystems*" which shows that temperature explains only 12% of the variance for CH_4 emissions in aquatic systems.

<u>Supplementary Note 4:</u>

Peeters and Hofmann speculated that the mesocosms may approach smaller CH_4 concentrations after a longer time period—We would welcome data to verify this speculation. Peeters and Hofmann also commented the central mesocosm was close to atmospheric saturation showing no indication of significant CH_4 production—This is consistent with what we discussed in the paper. Oxic methane production is a biological process that depends on certain set of environmental conditions, including nutrients and the relevant organisms. When nutrients and the related biological activities became severely limited in the central mesocosm, oxic methane production would become negligible as expected.

Supplementary Note 5:

We first explain why Peeters' and Hofmann's equation and their alternative oxic methane contribution to emission (OMC) estimations are incorrect, then we further clarify how we analysed DelSontro et al. 2018's data.

Peeters and Hofmann derived their version of OMC termed NOMC by comparing sediment and lake surface methane fluxes as NOMC = ($F_{surf,tot}$ – $F_{sed,tot}$)/$F_{surf,tot}$ (combining their equations of NOM and NOMC). By doing so, Peeters and Hofmann ignore any internal biochemical process of the aquatic methane production-consumption balance (e.g. *"This procedure neglects processes contributing to the mass balance…"*). In their calculations for additional lake estimates, Peeters and Hofmann further inadequately substitute the flux data $F_{surf,tot}$/$F_{litt,tot}$ with R_{CH4} values (*"Relative [CH_4] decrease/increase due to oxidation/production"*) taken from DelSontro et al. (Supplementary Table 8) resulting in the formula NOMC = (R_{CH4} – 1)/R_{CH4}. DelSontro et al. calculated R_{CH4} as the total change in methane concentration over the gas residence time (DelSontro et al. Table S8) relative to the background methane level along the transect (DelSontro et al. Supplementary Table 5)—this background methane was a combination of anoxic and oxic methane—as influenced by oxidation vs. production. In other words, DelSontro et al.'s calculations gave an indication of the dynamics of epilimnetic methane as influenced by the opposing processes of oxidation and production. Note that DelSontro et al. did not equate the background methane level to 'littoral methane'; to the contrary, their physical transport model predicts that littoral methane concentration decreases exponentially with distance from the shore due to dilution effect and emission loss, and in large lakes only negligible amounts of littoral methane (≤1.5 %) would reach the lake center. Actual measurements by DelSontro and colleagues showed that the concentration gradients largely deviated from this prediction (their Figs. 1, S4, S5, Table S5); accordingly, DelSontro et al. concluded that epilimnetic (oxic) methane production must be present. DelSontro et al. did not state that the observed concentrations (or background level) were due to littoral methane alone; yet, Peeters and Hofmann misrepresented the meaning of the "relative [CH_4] decrease/increase" (R_{CH4}) in DelSontro et al. and arbitrarily set a 100% baseline value for littoral methane, then expressed any relative change as NOMC (i.e. the '1' part of their equation). Therefore, the NOMC presented by Peeters and Hofmann is misleading and unjustified. We refer readers to DelSontro et al. 2018 for details. Accordingly, Peeters' and Hofmann's NOMC cannot be compared to our OMC estimates.

We would further clarify how we analysed DelSontro et al.'s data. In addition to concentration data, DelSontro et al. inferred epilimnetic methane production vs. oxidation from

methane carbon stable isotope data by assuming that methane $\delta^{13}C$ values lower than -54 ‰ indicates epilimnetic production, whereas δ values higher than -54 ‰ indicates oxidation (their Fig. 3). This interpretation was inadequate because the precise biochemical pathways, hence the isotopic signatures of oxically produced methane were unknown. As new information emerges (post publication of Günthel et al. 2019, e.g. Bizic et al. 2020, Günthel et al. 2020, Hartmann et al. 2020, Klintzsch et al. 2020), we now know that OMP can be linked to phototrophic fixation of inorganic carbon, which would likely yield a higher (heavier) $\delta^{13}C$ value than anoxically produced methane, thereby confounding isotopic signal of methane oxidation. To avoid uncertainties associated with the isotope data, we opted to estimate OMC based on methane concentrations along their sampling transect (data obtained via personal communications with Dr. DelSontro).

Discrepancy between observed methane concentrations and predicted methane concentrations based on their physical transport model along the transect represents contribution from oxic methane production, the rest was anoxic methane. We then extrapolated the transect data to the entire lake surface area (equivalent radius from DelSontro et al.) to obtain system-wide contributions of oxic and anoxic methane—Note this is vastly different from DelSontro et al. and Peeters and Hofmann, who only considered ‘relative [CH_4] decrease/increase’ along the transect measurements.

To explore how OMC varied with lake morphometry such as surface mixed-layer volume (Ꝟ) and littoral sediment area (A_{sed}), we calculated Ꝟ from data in DelSontro et al. (surface area, surface mixed layer depth), and we estimated A_{sed} based on a littoral sediment slope of 45°. Even when we change the sediment slope from 45° to 5° as suggested by Peeters and Hofmann, giving higher littoral sediment areas, the overall relationship between OMC and A_{sed} over Ꝟ only changes slightly (Fig. 1 in the main text).

Further, we would like to point out that Peeters and Hofmann excluded the source signature of oxically produced methane from all of their isotope considerations.

SUPPLEMENTARY REFERENCES

Althoff, F. et al. Abiotic methanogenesis from organosulphur compounds under ambient conditions. *Nat Commun* **5,** 4205 (2014). doi:10.1038/ncomms5205

Angel, R., Matthies, D. & Conrad, R. Activation of methanogenesis in arid biological soil crusts despite the presence of oxygen. *PLoS ONE* **6,** e20453 (2011). doi:10.1371/journal.pone.0020453

Angle, J.C. et al. Methanogenesis in oxygenated soils is a substantial fraction of wetland methane emissions. *Nat Commun* **8,** 1567 (2017). doi:10.1038/s41467-017-01753-4

Bizic, M. et al. Aquatic and terrestrial cyanobacteria produce methane. *Sci Adv* **6,** EAAX5343 (2020). doi:10.1126/sciadv.aax5343

Bogard, M. et al. Oxic water column methanogenesis as a major component of aquatic CH4 fluxes. *Nat Commun* **5,** 5350 (2014). doi:10.1038/ncomms6350

Carini, P., White, A., Campbell, E. & Giovannoni, S. J. Methane production by phosphate-starved SAR11 chemoheterotrophic marine bacteria. *Nat Commun* **5,** 4346 (2014). doi:10.1038/ncomms5346

Casper, P. Methane production in littoral and profundal sediments of an oligotrophic and a eutrophic lake. *Arch Hydrobiol Spec Issues Advanc Limnol* **48,** 253-259 (1996).

Casper, P., Furtado, A. L. S. & Adams, D. D. Biogeochemistry and diffuse fluxes of greenhouse gases (methane and carbon dioxide) and dinitrogen from the sediments of oligotrophic Lake Stechlin, Northern Germany. *Arch Hydrobiol Spec Issues Advanc Limnol* **58,** 53−71 (2003).

Casper, P. et al. Greenhouse gas cycling in aquatic ecosystems − methane in temperate lakes across an environmental gradient in northeast Germany. *Verh Int Ver Limnol* **29,** 564−566 (2005).

Conrad, R., Chan, O.-C., Claus, P. and Casper, P. Characterization of methanogenic Archaea and stable isotope fractionation during methane production in the profundal sediment of an oligotrophic lake (Lake Stechlin, Germany). *Limnol Oceanogr* **52,** 1393− 1406 (2007).

Damm, E. et al. Methane production in aerobic oligotrophic surface water in the central Arctic Ocean. *Biogeosciences* **7,** 1099–1108 (2010). doi:10.5194/bg-7-1099-2010

DelSontro, T., del Giorgio, P.A. & Prairie, Y.T. No longer a paradox: The interaction between physical transport and biological processes explains the spatial distribution of surface water methane within and across lakes. *Ecosystems* **21,** 1073–1087 (2018). doi:10.1007/s10021-017-0205-1

Donis, D., Flury, S., Stöckli, A. et al. Full-scale evaluation of methane production under oxic conditions in a mesotrophic lake. *Nat Commun* **8,** 1661 (2017). doi:10.1038/s41467-017-01648-4

Günthel, M. et al. Contribution of oxic methane production to surface methane emission in lakes and its global importance. *Nat Commun* **10,** 5497 (2019). doi:10.1038/s41467-019-13320-0

Günthel, M. et al. Photosynthesis-driven methane production in oxic lake water as an important contributor to methane emission. *Limnol Oceanogr* **9999,** 1-13 (2020). doi:10.1002/lno.11557

Gregg, M. C., D'Asaro, E. A., Riley, J. J., & Kunze, E. Mixing efficiency in the ocean. *Ann Rev Mar Sci* **10,** 443-473 (2018). doi:org/10.1146/annurev-marine-121916-063643

Grossart, H.-P., Frindte, K., Dziallas, C., Eckert, W. & Tang, K. W. Microbial methane production in oxygenated water column of an oligotrophic lake. *Proc Natl Acad Sci USA* **108,** 19657-19661 (2011). doi:10.1073/pnas.1110716108

Hartmann, J. F. et al. High spatiotemporal dynamics of methane production and emission in oxic surface water. *Environ Sci Technol* **54,** 1451-1463 (2020). doi:10.1021/acs.est.9b03182

Karl, D. et al. Aerobic production of methane in the sea. *Nature Geosci* **1,** 473–478 (2008). doi:10.1038/ngeo234

Keppler, F., Hamilton, J., Braß, M. & Röckmann, T. Methane emissions from terrestrial plants under aerobic conditions. *Nature* **439,** 187–191 (2006). doi:10.1038/nature04420

Khatun, S. et al. Aerobic methane production by planktonic microbes in lakes. *Sci Tot Environ* **696,** 133916 (2019). doi:10.1016/j.scitotenv.2019.133916

Khatun, S. et al. Linking stoichiometric organic carbon–nitrogen relationships to planktonic cyanobacteria and subsurface methane maximum in deep freshwater lakes. *Water* **12,** 402 (2020). doi:10.3390/w12020402

Kirillin, G., Grossart, H. P., & Tang, K. W. Modeling sinking rate of zooplankton carcasses: Effects of stratification and mixing. *Limnol Oceanogr* **57(3)**, 881-894 (2012). doi:org/10.4319/lo.2012.57.3.0881

Klintzsch, T. et al. Methane production by three widespread marine phytoplankton species: Release rates, precursor compounds, and potential relevance for the environment. *Biogeosciences* **16,** 4129-4144 (2019). doi:10.5194/bg-16-4129-2019

Klintzsch, T. et al. Effects of temperature and light on methane production of widespread marine phytoplankton. *J Geophys Res Biogeosci* **125,** e2020JG005793 (2020). doi:10.1029/2020JG005793

Lenhart, K. et al. Evidence for methane production by saprotrophic fungi. *Nat Commun* **3,** 1046 (2012). doi:10.1038/ncomms2049

Lenhart, K. et al. Evidence for methane production by the marine algae Emiliania huxleyi, *Biogeosciences* **13,** 3163–3174 (2016). doi:10.5194/bg-13-3163-2016

Leon-Palmero, E., Contreras-Ruiz, A., Sierra, A., Morales-Baquero, R. & Reche, I. Dissolved CH4 coupled to photosynthetic picoeukaryotes in oxic waters and to cumulative chlorophyll a in anoxic waters of reservoirs, *Biogeosciences* **17,** 3223–3245 (2020). doi:10.5194/bg-17-3223-2020

Li, W. et al. Methane production in the oxygenated water column of a perennially ice-covered Antarctic lake. *Limnol Oceanogr* **65,** 143-156 (2020). doi:10.1002/lno.11257

MacIntyre, S. et al. Buoyancy flux, turbulence, and the gas transfer coefficient in a stratified lake. *Geophys Res Lett* **37,** L24604. doi:10.1029/2010GL044164

McGinnis, D. F. et al. Enhancing surface methane fluxes from an oligotrophic lake: exploring the microbubble hypothesis. *Environ Sci Technol* **49,** 873-880 (2015). doi:10.1021/es503385d

Messenger, D. J., McLeod, A. R. & Fry, S. C. The role of ultraviolet radiation, photosensitizers, reactive oxygen species and ester groups in mechanisms of methane formation from pectin. *Plant Cell Environ* **32,** 1-9 (2009). doi:10.1111/j.1365-3040.2008.01892.x

Peeters, F., Fernandez, E. J. & Hofmann, H. Sediment fluxes rather than oxic methanogenesis explain diffusive CH4 emissions from lakes and reservoirs. *Sci Rep* **9,** 243 (2019). doi:10.1038/s41598-018-36530-w

Perez-Coronel, E. & Beman, J. M. Biogeochemical and omic evidence for multiple paradoxical methane production mechanisms in freshwater lakes. Preprint at *bioRxiv* (2020). doi:10.1101/2020.07.28.225276

Repeta, D. et al. Marine methane paradox explained by bacterial degradation of dissolved organic matter. *Nat Geosci* **9,** 884–887 (2016). doi:10.1038/ngeo2837

Tang, K. W. McGinnis, D. F., Frindte, K., Brüchert, V. & Grossart, H.-P. Paradox reconsidered: Methane oversaturation in well-oxygenated lake waters. *Limnol Oceanogr* **59,** 275-284 (2014). doi:10.4319/lo.2014.59.1.0275

Tsunogai, U. et al. Dual stable isotope characterization of excess methane in oxic waters of a mesotrophic lake. *Limnol Oceanogr* **9999,** 1-16 (2020). doi:10.1002/lno.11566

Wang, Q., Dore, J. E. & McDermott, T. R. Methylphosphonate metabolism by Pseudomonas sp. populations contributes to the methane oversaturation paradox in an oxic freshwater lake. *Environ Microbiol* **19,** 2366-2378 (2017). doi:10.1111/1462-2920.13747

White, A. E., Karl, D. M., Björkman, K. M., Beversdorf, L. J. & Letelier, R. M. Phosphonate metabolism by Trichodesmium IMS101 and the production of greenhouse gases. *Limnol Oceanogr* **55,** 1755-1767 (2010). doi:10.4319/lo.2010.55.4.1755

Yao, M. C, Henny, C. & Maresca, J. A. Freshwater bacteria release methane as a by-product of phosphorus acquisition. *J Appl Environ Microbiol* **82,** 6994-7003 (2016). doi:10.1128/AEM.02399-1

Ye, W. W., Wang, X. L., Zhang, X. H. & Zhang, G. L. Methane production in oxic seawater of the western North Pacific and its marginal seas. *Limnol Oceanogr* **9999,** 1-14 (2020). doi:10.1002/lno.11457

Yvon-Durocher, G. et al. Methane fluxes show consistent temperature dependence across microbial to ecosystem scales. *Nature* **507,** 488–491 (2014). doi:10.1038/nature13164
